# Validation of Urban Parks for Physical Activity Enhancement in Community-Dwelling Older Adults: A Developing Country Experience

**DOI:** 10.7759/cureus.92855

**Published:** 2025-09-21

**Authors:** Lilian Solis-Navarro, Rodrigo Torres-Castro, Edgardo Opazo-Díaz, Alfonsina Puppo-Stuardo, Sofía Dávila-Oña, Marisol Barros-Poblete, Matías Otto-Yáñez, Ane Arbillaga-Etxarri, Elena Gimeno-Santos, Mercé Sitjà-Rabert, Laura M Pérez-Bazán

**Affiliations:** 1 Department of Physical Therapy, University of Chile, Santiago, CHL; 2 Research, Laboratorio de Ecosistemas Urbanos, Santiago, CHL; 3 Physiotherapy, Pontificia Universidad Católica del Ecuador, Manabí, ECU; 4 Doctoral Program in Medical Sciences, Universidad Austral, Valdivia, CHL; 5 Medicine, Grupo de Investigación en Salud, Funcionalidad y Actividad Física (GISFAF) Kinesiología, Facultad de Ciencias de la Salud, Universidad Autónoma de Chile, Santiago, CHL; 6 Department of Physical Therapy, Faculty of Health Sciences, University of Deusto, Donostia, San Sebastián, ESP; 7 Pulmonology, Hospital Clinic de Barcelona, Barcelona, ESP; 8 Faculty of Health Sciences, Universitat Ramon Llull, Barcelona, ESP; 9 Research, RE-FiT Barcelona Research Group, Parc Sanitari Pere Virgili Hospital and Vall d'Hebron Institut de Recerca (VHIR), Barcelona, ESP

**Keywords:** older adults, oxygen consumption, public parks, urban built environment, walking

## Abstract

Background and objective

Built-environment guidance rarely translates into practice-ready walking prescriptions for older adults. In light of this, we calibrated graded, cadence-based urban park trails to generate target intensities and expected physiological responses to inform primary care and municipal signage.

Methods

Community-dwelling adults aged ≥60 years completed three predefined trails (low-, medium-, and high-intensity). Oxygen uptake (VO₂), minute ventilation (VE), and heart rate (HR) were recorded with a portable metabolic system and an optical HR sensor; cadence was metronome-guided to reach target intensities. Outcomes included VO₂, VE, HR, energy expenditure per ~12-15-minute bout, and perceived exertion.

Results

Physiological responses exhibited a graded, dose-responsive profile across trails. Mean VO₂, VE, HR, and energy expenditure increased from low to high, while walking time remained feasible for a brief bout. We provide practice-ready parameters (distance and cadence bands with expected intensity ranges) for implementation. No adverse events occurred.

Conclusions

Calibrated park trails can operationalize safe, scalable walking prescriptions for older adults. These translational outputs (distance and cadence ranges) are directly usable by clinicians and municipalities. Future evaluations should assess uptake, adherence, and health outcomes in real-world settings.

## Introduction

The global population of older adults is experiencing unprecedented growth. Between 2015 and 2050, the proportion of individuals aged 60 years or older is expected to nearly double, increasing from 12% to 22% [1]. This demographic phenomenon has gained significant relevance, particularly in the Latin American context, which has strongly influenced public policies aimed at promoting healthy aging [2,3]. The aging of the population is accompanied by a rising prevalence and incidence of noncommunicable diseases (NCDs) [4]. This growing burden strains healthcare resources and infrastructure, affects quality of life, and challenges the sustainability of health systems [4]. Sedentary behavior, characterized by prolonged physical inactivity, is a key modifiable risk factor that exacerbates the global burden of NCDs and contributes to declining functional capacity and overall well-being among older adults [5].

Regular physical activity is widely recognized as an effective strategy to counter the detrimental effects of sedentary behavior. It provides both preventive and therapeutic benefits by enhancing cardiovascular health, preserving musculoskeletal function, and improving mental well-being in older populations [6,7]. Clinical evidence also shows that physical activity helps slow the deterioration of muscle strength and cardiorespiratory fitness, preserves functional capacity, and contributes to the management of chronic conditions [8,9]. From a sociological perspective, physical activity fosters enjoyment of the body in later life, promotes overall well-being, strengthens social connections, mitigates loneliness, and supports a higher quality of life [10,11].

From a health-promotion standpoint, walkability and access to safe, pleasant parks are modifiable social and environmental determinants that shape older adults’ behavior and safely achievable intensity. Walking is widely adopted, and built-environment features - such as park availability, trail design, slope, surface, and shade - govern walking behavior and achievable intensity [12,13]. Additionally, models such as the Capability Opportunity Motivation-Behaviour (COM-B) model suggest that older adults’ participation in physical activity depends on their capability (both physical and psychological), opportunity (arising from physical and social environments), and motivation (automatic and reflective) [14]. Understanding the specific factors that shape these three components in later life can guide the development of more effective interventions [15]. 

To generate practice- and policy-relevant guidance, we conducted a proof-of-concept, cross-sectional, repeated-measures, field-based validation of graded, cadence-calibrated urban park trails in older adults using portable metabolic measurements. We prespecified cadence targets for each trail, documented place descriptors (including distance, slope, surface, and shade/amenities), and quantified in-situ physiological responses during standardized walking. We then translated these data into simple parameters (distance, cadence, and calories) suitable for park signage and primary care counseling. Our objective was to validate urban training routes in public parks of varying intensities by assessing the physiological responses of older adults while walking these trails.

## Materials and methods

Study design

We conducted a cross-sectional, repeated-measures, proof-of-concept study between March and May 2024 in a metropolitan public park in Santiago (Parque Los Reyes), Chile.

Ethical considerations

The study was approved by the Ethics Committee of the Faculty of Medicine, University of Chile (approval No. 158-2023). All participants provided written informed consent. Reporting followed the Strengthening the Reporting of Observational Studies in Epidemiology (STROBE) guidelines [16].

Participants

We included adults aged ≥60 years who walked independently. We excluded individuals with acute respiratory disease within the past 30 days, those with musculoskeletal or neurological conditions that impair walking, and those who were unable to understand or follow instructions. Individuals with chronic respiratory disease were excluded if they had experienced a recent exacerbation.

Setting and protocol

**Figure 1:**
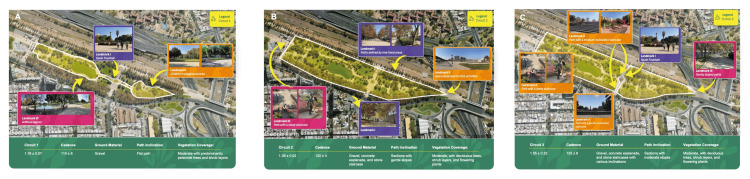
Place-based mapping of the three graded walking trails used in the study (A) Low-intensity, (B) medium-intensity, and (C) high-intensity trails within a metropolitan public park in Santiago, Chile. Each panel displays the trail polyline (white), direction of travel (yellow arrows), and representative landmarks; the green footer summarizes key place descriptors, including distance, achieved cadence, ground material, path inclination, and vegetation/shade. Mean trail distances were 1.19 km (low), 1.39 km (medium), and 1.55 km (high); achieved cadences were ~110, 120, and ~125 steps·min⁻¹, respectively. Scale bar and north arrow are provided in each panel Background basemap: Google Earth imagery (©Google; imagery/data providers as credited in Google Earth). Original content: all overlays/annotations, and ground photographs were created and captured by the authors (research team), who hold the copyright to these elements. Permission/license: static Google Earth imagery is used under Google Maps/Earth permissions for media (static screenshot with required attribution); therefore, no additional permission is required

A multidisciplinary team (physiotherapists, pulmonologists, sport-science experts, and urban planners) identified three walking trails within the park (Figure 1) representing low, moderate, and high intensity based on place descriptors (distance, mean/maximum slope, surface type, shade/amenities), following recommendations from the WHO, the Urban Training project, and the Healthy Cities movement [17].

Target cadences were 110, 120, and 130 steps·min⁻¹. Participants completed all three trails once in randomized order. Cadence was guided by a metronome app and verbal cues, with 60-s step counts verifying adherence. Before testing, anthropometrics were measured using a stadiometer-equipped scale, and heart rate (HR) and gas analysis devices were fitted. Each 12-15-minute trail was followed by a 30-minute rest.

Sample size

This study was conceived as a proof-of-concept, repeated-measures field validation focused on within-subject physiological responses across three graded trails. Assuming a standard deviation (SD) of oxygen uptake (VO₂) of 3 ml/min/kg [18], a detectable within-subject difference of 3.0 mL·kg⁻¹·min⁻¹, with α = 0.05 and power = 0.80 (two-sided), 10 subjects were required to detect a minimum difference of 3 units in VO2. A repeated-measures design increases precision and power with fewer participants, making it an appropriate choice for feasibility testing in real-world settings. We anticipated ≤20% attrition, which did not occur.

Measurements

We recorded sex, weight, height, and BMI. A portable gas analyzer (MetaMax 3B2R, Cortex, Leipzig, Germany) was used to determine VO₂, minute ventilation (VE), carbon dioxide production (VCO₂), respiratory exchange ratio (RER = VCO₂/VO₂), and oxygen pulse (O₂-pulse). HR was monitored using an optical armband device (Polar Sense, Polar Electro, Helsinki, Finland).

Statistical analysis

Analyses were conducted using IBM SPSS Statistics v23.0 (IBM Corp., Armonk, NY). Sociodemographic variables were summarized descriptively: qualitative variables as n (%) and quantitative variables as mean ± SD (or median [interquartile range, IQR] when non-normal). Primary comparisons across the three trails used one-way repeated-measures ANOVA. Mauchly’s test assessed sphericity; Greenhouse-Geisser corrections were applied when violated. Pairwise comparisons employed the Bonferroni adjustment. A two-sided p<0.05 was considered statistically significant.

## Results

A total of 10 participants (eight women) completed the exercise trails in this study. The mean age of the cohort was 65.3 ± 3.3 years, the mean weight was 63.2 ± 4.1 kilograms, height was 1.57 ± 0.08 meters, and BMI was 25.7 ± 2.0 kg/m², respectively (Table 1). The distance extended from 1.19 ± 0.07 km at low intensity to 1.55 ± 0.11 km at high intensity (p<0.001). The VO_2_, VCO_2_, RER, and VE increased progressively with circuit intensity (Table 2).

**Table 1:** Baseline demographic and anthropometric characteristics of participants BMI: body mass index

Subject	Sex	Age (years)	Height (m)	Weight (kg)	BMI (kg/m²)
S1	Female	60	1.5	62.5	27.8
S2	Female	66	1.56	65	26.7
S3	Male	63	1.75	63.6	20.8
S4	Male	67	1.65	71.3	26.2
S5	Female	69	1.52	60.5	26.2
S6	Female	60	1.57	67.3	27.3
S7	Female	69	1.58	62.5	25
S8	Female	67	1.5	61	27.1
S9	Female	68	1.47	56.2	26
S10	Female	63	1.6	62.2	24.3

**Table 2:** Physiological responses and exercise characteristics across walking trails of different intensities The values are expressed in mean ± standard deviation EE: energy expenditure; HR: heart rate; O_2_ pulse: oxygen pulse; RER: respiratory exchange ratio; RR: respiratory rate; VCO_2_: carbon dioxide production; VE: minute ventilation; VO_2_: oxygen consumption

	Low intensity	Medium intensity	High intensity	P-value: between-group differences	Low vs. medium intensity	Low vs. high intensity	Medium vs. high intensity
VO_2 _(L/min)	0.79 ± 0.10	0.96 ± 0.13	1.10 ± 0.19	0.001	0.001	<0.001	0.013
VCO_2_ (L/min)	0.70 ± 0.10	0.87 ± 0.13	1.04 ± 0.18	<0.001	0.001	<0.001	0.009
RER	0.86 ± 0.05	0.90 ± 0.05	0.92 ± 0.04	0.014	0.312	0.015	0.504
VO_2_/Kg (ml/kg/min)	12.7 ± 0.95	15.0 ± 1.7	17.3 ± 1.98	<0.001	0.004	<0.001	0.005
VE (L/min)	24.7 ± 2.15	30.3 ± 4.6	37.0 ± 6.7	0.001	0.019	<0.001	0.004
HR (bpm)	109 ± 16	116 ± 12	122 ± 15	0.054	0.303	0.050	0.076
O_2_pulse (ml/bpm)	7.49 ± 1.40	8.34 ± 1.36	9.18 ± 1.86	0.027	0.077	0.018	0.032
RR (breathe/min)	27 ± 5	30 ± 7	33 ±7	0.004	0.327	0.006	0.010
EE (kcal/h)	235 ± 30	281 ± 37	327 ± 50	<0.001	0.003	<0.001	0.004
Distance (km)	1.19 ± 0.07	1.39 ± 0.02	1.55 ± 0.11	<0.001	<0.001	<0.001	0.011
Cadence (steps/min)	110 ± 6	120 ± 4	125 ± 6	0.001	0.003	<0.001	0.023

HR showed a trend of increasing with intensity, although the changes were not statistically significant (p = 0.054). O_2_ pulse significantly increased from 7.49 ± 1.40 ml/bpm at low intensity to 9.18 ± 1.86 ml/bpm at high intensity (p = 0.027). The respiratory rate increased from 27 ± 5 breaths/min at low intensity to 33 ± 7 breaths/min at high intensity (p = 0.004). Energy expenditure reflected significant rises with each increase in circuit intensity, with values of 235 ± 30 kcal/h at low intensity and 327 ± 50 kcal/h at high intensity (p<0.001).

No adverse events were reported during the study, and all participants completed the walking trails without complications. The protocol was well tolerated, reinforcing its feasibility for future large-scale implementations.

## Discussion

This park-based, proof-of-concept study demonstrated that three walking trails within a public park elicited a clear, graded physiological load in older adults, with VO₂ increasing across trail intensities. The characteristics that determined the change in the physiological response to exercise were the presence of slopes in the circuit and the intensity of the walking cadence. This pattern is consistent with studies showing that built-environment features can facilitate higher-intensity walking opportunities in older adults [19,20] and with exercise physiology evidence in this population [6,7]. In line with this framework, Arbillaga-Etxarri et al. reported dose-responsive increases in energy expenditure and physiological load with graded urban-training circuits in chronic disease populations [21]. While these acute responses indicate a meaningful physiological challenge, further research is needed to determine whether repeated exposure to these intensities leads to long-term adaptations in cardiovascular and muscular capacity.

The validation and use of walking trails embedded within urban public spaces highlight how intentional place design can serve as a lever for increasing physical activity in diverse populations, including those with chronic conditions [13,22]. Studies indicate that when trails are integrated into accessible, attractive, and socially safe environments, daily activity levels can rise, even months after structured programs end. Although improvements in exercise capacity are not consistently demonstrated, the persistence of park-based physical activity supports a population-level strategy with potential long-term health and equity benefits, particularly in settings with limited recreational infrastructure.

Implementing calibrated circuits in urban parks is a pragmatic public health strategy for aging populations with rising non-communicable disease burdens. Such programs can be adapted and scaled across urban contexts, providing accessible tools to support health promotion [23]. Beyond physical benefits, these environments may also foster mental and social well-being by providing spaces for exercise, relaxation, and social interaction, elements that are particularly relevant for older adults [24]. In Chile, where socioeconomic inequalities affect access to quality parks, our results are particularly relevant. De la Barrera et al. [25] emphasize the role of urban parks in mitigating adverse environmental conditions and delivering ecosystem services that support well-being and public health. At the same time, Henderson [26] highlights their contribution to promoting physical activity and health. Our findings add that calibrated, park-based circuits can reinforce these benefits by facilitating structured yet accessible activity in community settings.

These aspects should also be considered when interpreting the findings. Lifestyle habits, including sedentary behavior, smoking, and alcohol consumption, as well as previous or current occupational activities, may influence the physiological response and overall outcomes [27]. The heterogeneity among older adults - where some remain highly active through ongoing work or daily routines, while others are more sedentary - could partly explain differences in performance and adaptation [27]. In addition, factors such as cognitive status, lack of time, the need for activities to be free or low-cost, accessibility, and transportation to the location, and even weather conditions may further affect participation and outcomes [27]. Future studies should incorporate these variables to provide a more comprehensive understanding of the factors that shape responses to physical activity interventions in this population. Future studies should investigate how specific design features (e.g., distance, slope, surface, shade/amenities) affect achieved intensity and activity type, and whether repeated exposure to these trails enhances cardiorespiratory fitness, metabolic function, and mental well-being.

This study has several limitations, including a relatively small sample size and the geographic concentration of participants, which may limit the generalizability of our results. Additionally, individual variability in exercise capacity due to pre-existing health conditions was not considered, which could influence the physiological responses observed. Additionally, gender disparity and the lack of comparisons with other parks should be acknowledged as limitations that may restrict the generalizability of the findings. Furthermore, the extrapolation of these results to other global contexts is limited by factors such as climate variations, differences in urban infrastructure, and safety conditions, which may impact the feasibility and effectiveness of implementing similar trails in different regions. Another limitation is that the physiological variables were not normalized to participants’ maximal capacities, making direct comparisons between individuals more challenging and potentially influencing the interpretation of exercise intensity responses.

## Conclusions

Urban training trails of varying intensity in public parks elicit physiological responses and increased energy expenditure in older adults, demonstrating their potential as accessible and cost-effective interventions to promote physical activity and healthy aging. These parameters can be readily translated into park signage and primary care counseling using simple distance-cadence cues. The approach is low-cost and scalable, requiring minimal infrastructure and staff training, which facilitates uptake in resource-constrained urban settings. Embedding calibrated trails within existing municipal parks can promote equitable access to safe, structured walking opportunities for older residents. Future multi-site, longitudinal evaluations should examine real-world uptake, adherence, and sustained effects on functional capacity and well-being.

## References

[1] (2025). World Health Organization. ageing and health. https://www.who.int/news-room/fact-sheets/detail/ageing-and-health.

[2] Santamaria-Garcia H, Sainz-Ballesteros A, Hernandez H (2023). Factors associated with healthy aging in Latin American populations. Nat Med.

[3] Keating NC, Rodríguez Mañas L, De Francisco A (2021). Toward healthy aging in Latin America and the Caribbean: leaving no one behind?. Rev Panam Salud Publica.

[4] Asogwa OA, Boateng D, Marzà-Florensa A, Peters S, Levitt N, van Olmen J, Klipstein-Grobusch K (2022). Multimorbidity of non-communicable diseases in low-income and middle-income countries: a systematic review and meta-analysis. BMJ Open.

[5] Pierre J, Collinet C, Schut PO, Verdot C (2022). Physical activity and sedentarism among seniors in France, and their impact on health. PLoS One.

[6] Mora JC, Valencia WM (2018). Exercise and older adults. Clin Geriatr Med.

[7] McPhee JS, French DP, Jackson D, Nazroo J, Pendleton N, Degens H (2016). Physical activity in older age: perspectives for healthy ageing and frailty. Biogerontology.

[8] Pedersen BK, Saltin B (2015). Exercise as medicine - evidence for prescribing exercise as therapy in 26 different chronic diseases. Scand J Med Sci Sports.

[9] Izquierdo M, Merchant RA, Morley JE (2021). International Exercise Recommendations in Older Adults (ICFSR): expert consensus guidelines. J Nutr Health Aging.

[10] Langhammer B, Bergland A, Rydwik E (2018). The importance of physical activity exercise among older people. Biomed Res Int.

[11] Lautenschlager NT, Almeida OP, Flicker L, Janca A (2004). Can physical activity improve the mental health of older adults?. Ann Gen Hosp Psychiatry.

[12] Ng YL, Hill KD, Levinger P, Burton E (2021). Effectiveness of outdoor exercise parks on health outcomes in older adults-a mixed-methods systematic review and meta-analysis. J Aging Phys Act.

[13] Arbillaga-Etxarri A, Gimeno-Santos E, Barberan-Garcia A (2018). Long-term efficacy and effectiveness of a behavioural and community-based exercise intervention (Urban Training) to increase physical activity in patients with COPD: a randomised controlled trial. Eur Respir J.

[14] Michie S, van Stralen MM, West R (2011). The behaviour change wheel: a new method for characterising and designing behaviour change interventions. Implement Sci.

[15] Meredith SJ, Cox NJ, Ibrahim K (2023). Factors that influence older adults' participation in physical activity: a systematic review of qualitative studies. Age Ageing.

[16] von Elm E, Altman DG, Egger M, Pocock SJ, Gøtzsche PC, Vandenbroucke JP (2007). The Strengthening the Reporting of Observational Studies in Epidemiology (STROBE) statement: guidelines for reporting observational studies. Lancet.

[17] (2025). World Health Organization. Health as the pulse of the new urban agenda: The United Nations Conference on Housing and Sustainable Urban Development. Quito, October.

[18] Castro AA, Porto EF, Iamonti VC, de Souza GF, Nascimento OA, Jardim JR (2013). Oxygen and ventilatory output during several activities of daily living performed by COPD patients stratified according to disease severity. PLoS One.

[19] Talal ML, Santelmann MV (2021). Visitor access, use, and desired improvements in urban parks. Urban For Urban Green.

[20] Zhang J, Cheng Y, Zhao B (2021). How to accurately identify the underserved areas of peri-urban parks? An integrated accessibility indicator. Ecol Indic.

[21] Arbillaga-Etxarri A, Torrent-Pallicer J, Gimeno-Santos E (2016). Validation of walking trails for the Urban Training™ of chronic obstructive pulmonary disease patients. PLoS One.

[22] Torres-Castro R, Vilaró J, Martí JD (2019). Effects of a combined community exercise program in obstructive sleep apnea syndrome: a randomized clinical trial. J Clin Med.

[23] Poppe L, Van Dyck D, De Keyser E, Van Puyvelde A, Veitch J, Deforche B (2023). The impact of renewal of an urban park in Belgium on park use, park-based physical activity, and social interaction: a natural experiment. Cities.

[24] Kaczynski AT, Henderson KA (2007). Environmental correlates of physical activity: a review of evidence about parks and recreation. Leis Sci.

[25] Barrera F de la, Henriquez C, Ruiz V, Inostroza L (2019). Urban parks and social inequalities in the access to ecosystem services in Santiago, Chile. IOP Conf Ser Mater Sci Eng.

[26] Henderson KA (2006). Urban parks and trails and physical activity. Ann Leis Res.

[27] Yang Y, Gao Y, An R, Wan Q (2024). Barriers and facilitators to exercise adherence in community-dwelling older adults: a mixed-methods systematic review using the COM-B model and Theoretical Domains Framework. Int J Nurs Stud.

